# Invasion genetics from eDNA and thousands of larvae: A targeted metabarcoding assay that distinguishes species and population variation of zebra and quagga mussels

**DOI:** 10.1002/ece3.4985

**Published:** 2019-03-04

**Authors:** Nathaniel T. Marshall, Carol A. Stepien

**Affiliations:** ^1^ Genetics and Genomics Group, Department of Environmental Sciences The University of Toledo Toledo Ohio; ^2^ Genetics and Genomics Group NOAA Pacific Marine Environmental Laboratory Seattle, Washington

**Keywords:** *Dreissena*, environmental DNA, Great Lakes, high‐throughput sequencing, invasive species

## Abstract

Identifying species and population genetic compositions of biological invasions at early life stages and/or from environmental (e)DNA using targeted high‐throughput sequencing (HTS) metabarcode assays offers powerful and cost‐effective means for early detection, analysis of spread patterns, and evaluating population changes. The present study develops, tests, and applies this method with a targeted sequence assay designed to simultaneously identify and distinguish between the closely related invasive Eurasian zebra and quagga mussels (*Dreissena polymorpha* and *D. rostriformis*) and their relatives and discern their respective population genetic patterns. Invasions of these dreissenid mussel species have markedly changed freshwater ecosystems throughout North America and Europe, exerting severe ecological and economic damage. Their planktonic early life stages (eggs and larvae) are morphologically indistinguishable, yet each species exerts differential ecological effects, with the quagga often outcompeting the zebra mussel as adults. Our targeted assay analyzes genetic variation from a diagnostic sequence region of the mitochondrial (mt)DNA cytochrome oxidase I (COI) gene, to assess temporal and spatial inter‐ and intra‐specific genetic variability. The assay facilitates analysis of environmental (e)DNA from water, early life stages from thousands of individuals, and simultaneous analysis of 50–100 tagged field‐collected samples. Experiments evaluated its accuracy and performance using: (a) mock laboratory communities containing known DNA quantities per taxon, (b) aquaria with mixed‐species/haplotype compositions of adults, and (c) field‐collected water and plankton versus traditional sampling of adult communities. Results delineated species compositions, relative abundances, and population‐level diversity differences among ecosystems, habitats, time series, and life stages from two allopatric concurrent invasions in the Great Lakes (Lake Erie) and the Hudson River, which had separate founding histories. Findings demonstrate application of this targeted assay and our approach to accurately and simultaneously discern species‐ and population‐level differences across spatial and temporal scales, facilitating early detection and ecological understanding of biological invasions.

## 1 INTRODUCTION

**Figure 1:**
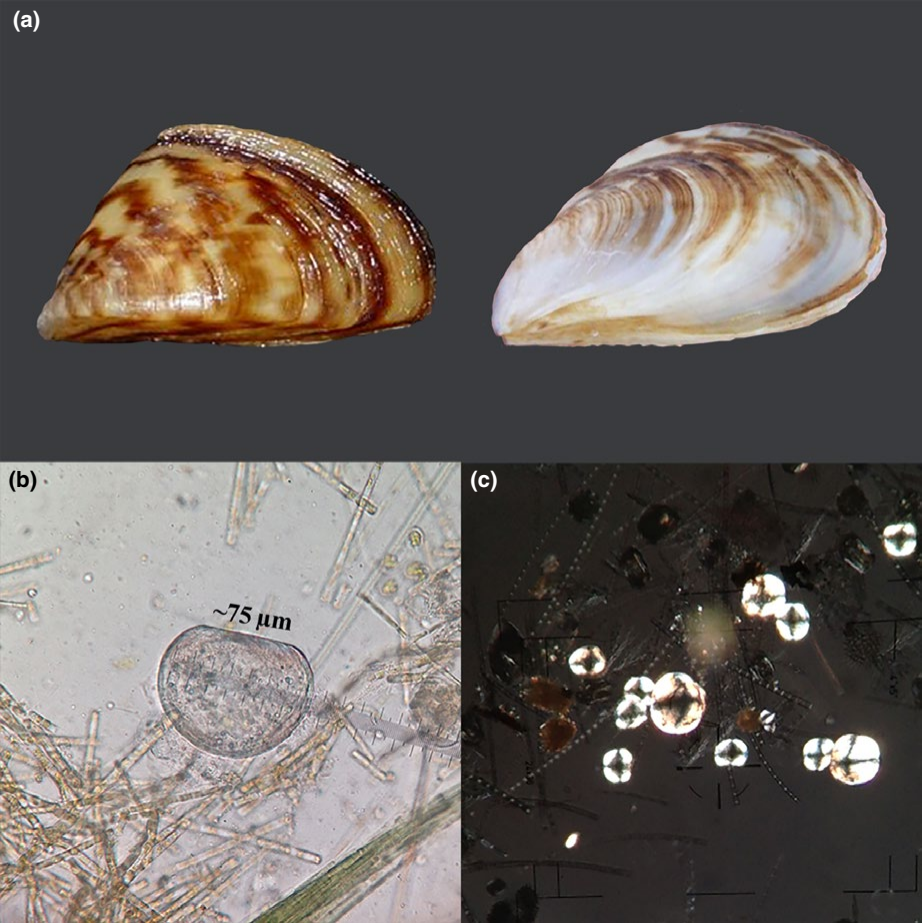
(a) Adult zebra mussel *Dreissena polymorpha* (left) and quagga mussel *D. rostriformis* (right) (each specimen about 2.0 cm in length, (photographs not copyrighted and used courtesy of USGS NAS; https://nas.er.usgs.gov/)). *Dreissena* veliger larvae from our plankton samples (b) under compound light microscope (single larva) and (c) using cross‐polarized light (multiple larvae) taken by NTM in this study

The growing global prevalence of invasive species profoundly shapes contemporary ecosystems (Pimentel, 2014; Simberloff, 2013) and negatively affects commerce (Keller, Lodge, Lewis, & Shogren, 2009; Pimentel, Zuniga, & Morrison, 2005). Ecological effects of invasive species range from competition with native species for food and space, alterations in habitat structure, and spread of pathogens and parasites (Gallardo, Clavero, Sánchez, & Vilà, 2016; Kvach & Stepien, 2008; Simberloff, 2013). The North American Laurentian Great Lakes is one of the most serious examples of a heavily invaded ecosystem, where >184 aquatic invasive species (AIS) have established (NOAA, 2018). Of these, the invasions of two closely related bivalve dreissenid mussels from Eurasia (Figure 1)—the zebra mussel *Dreissena polymorpha *(Pallas, 1771) and the quagga mussel *D. rostriformis* (Deshayes, 1838)—are regarded to have exerted some of the greatest ecological and economic impacts. Dreissenids are extensive biofoulers, prolific filterers, and ecosystem “engineers” that convert soft to hard benthos, often hosting entire communities of accompanying organisms on their shell surfaces and interstices (Stepien et al., 2013; Ward & Ricciardi, 2007).

Over the past 300 years, the zebra mussel has spread throughout much of Europe from its native Ponto Caspian region, and the quagga mussel for the last 130 years (van der Velde, Rajagopal, & Bij de Vaate, 2010; Zhulidov et al., 2010). The first North American appearance of dreissenids was reported in 1986, with the zebra mussel's discovery in Lake St. Clair of the Great Lakes, attributed to ballast water released from one or more trans‐oceanic vessels (Hebert, Muncaster, & Mackie, 1989). This was followed by the 1989 detection of the quagga mussel in Lake Erie (May & Marsden, 1992).

Zebra mussel populations in the Great Lakes initially increased very rapidly, whereas the quagga mussel remained rare for several years, being primarily confined to deeper water habitats in eastern Lake Erie (Karatayev et al., 2014; Stepien, Hubers, & Skidmore, 1999). Near‐complete replacement of the zebra mussel by the quagga mussel since has occurred throughout the central and eastern Lake Erie basins, with the western basin now harboring a mixed‐species community, where shallower waters provide refugia for the zebra mussel (Karatayev et al., 2014). Lake Erie has the longest history of harboring established populations of both species (Benson, 2013), constituting a focal area for this study.

In comparison, the zebra mussel first invaded the Hudson River in 1991 (Strayer et al., 1996), followed by the quagga mussel in 2008 (Strayer & Malcom, 2013). This zebra mussel population initially grew very rapidly, accounting for >50% of the benthic biomass by 1992 (Strayer et al., 1996). Genetic analyses using microsatellites showed that zebra mussel populations from the Hudson River and Lake Erie significantly differed, implicating different founding origins (Brown & Stepien, 2010; Stepien et al., 2013). By 2010, the quagga mussel had spread throughout the Hudson River's entire freshwater reach, yet has remained only a small fraction (<10%) of the dreissenid community (Strayer & Malcom, 2013; Strayer, pers. comm.). The respective histories and relative compositions of the two species in the Hudson River and Lake Erie thus comprise an ecological contrast for the present investigation.

Early detection and continued monitoring of dreissenid introductions and spread patterns are high management agency priorities in the United States (NOAA, 2018; USGS, 2018), Canada (DFO, 2018; MNRF, 2018), and Europe (van der Velde et al., 2010; Zhulidov et al., 2010). Ecological effects of dreissenid invasions largely depend on their respective species composition, time elapsed since their establishment, population density, and hydrological characteristics of the waterbody (Benson, 2013). Predicting their ecological impacts thus relies on up‐to‐date information about their relative species composition and abundances. However, these sister taxa are difficult to distinguish to species as adults, sometimes even by trained malacologists, and cannot be differentiated at early life stages (as eggs or larvae; Nichols & Black, 1994; Claxton & Boulding, 1998).

Dreissenids are prolific, reproducing multiple times each season, with >1,000,000 eggs per female zebra mussel (Borcherding, 1991), and have free‐swimming veliger larvae that widely disperse (Ackerman, Sim, Nichols, & Claudi, 1994; Figure 1b–c). Adult mussels attach in tremendous numbers to vessels, tackle, and drifting objects and can survive out of water for a week or longer (Collas et al., 2014; Stepien et al., 2013); they thus are readily transported among bodies of water. New introductions often pose significant biosecurity risks (Robinson, Burgman, & Cannon, 2011), since dreissenid populations often are not identified until they are already established and widespread, and by then are difficult (or impossible) to eradicate (Zaiko, Minchin, & Olenin, 2014). Genetic tools that effectively identify and diagnose dreissenids at early life stages (from plankton) and at low densities (e.g., environmental (e)DNA from water samples) would significantly improve timely detection, identification, and monitoring.

Over the past two decades, identifying individual species from their DNA via “barcoding” (see Hebert, Cywinska, Ball, & deWaard, 2003) has increased diagnosis of taxa (Radulovici, Archambault, & Dufresne, 2010). Analysis of eDNA shed into water (via urine, waste, mucus, sloughed cells) has facilitated early detection of some AIS, but mostly has been limited to the detection of single species (Darling & Mahon, 2011; Ficetola, Miaud, Pompanon, & Taberlet, 2008; Jerde, Mahon, Chadderton, & Lodge, 2011). For example, eDNA was used to discover the quagga mussel in the Rhine River harbor of Basel, Switzerland, which had been missed by traditional monitoring (De Ventura, Kopp, Seppälä, & Jokela, 2017). Similarly, the zebra mussel was discerned from eDNA in the Red River off Lake Winnipeg, Canada, in 2014, a year before the first adult was located (Gingera, Bajno, Docker, & Reist, 2017). Although those studies detected dreissenids (De Ventura et al., 2017; Egan et al., 2015; Gingera et al., 2017; Ram et al., 2011), their methodology was unable to differentiate between zebra and quagga mussel species using a single assay and could not address population‐level variation (e.g., haplotypes). Targeted metabarcoding assays with high‐throughput sequencing (HTS) and bioinformatic processing can be designed to simultaneously resolve entire communities to species level, as well as distinguish population genetic variation (Elbrecht, Vamos, Steinke, & Leese, 2018; Parsons, Everett, Dahlheim, & Park, 2018; Sigsgaard et al., 2016; Stepien, Snyder, & Elz, 2019).

### 1.1 Research objective and applications

The research objective was to develop, test, and apply a new targeted HTS metabarcode assay that simultaneously identifies and differentiates among all six *Dreissena *species (see Stepien et al., 2013; Marshall & Stepien, 2019), including zebra and quagga mussels, and discerns their respective population‐level variability (i.e., haplotype representation and relative abundances) at all life stages. Experiments tested the assay's accuracy and performance on mixed communities of zebra and quagga mussel species and their haplotypes in: (a) a series of mock laboratory communities containing known quantities of DNA, (b) aquarium experiments, and (c) field‐collected eDNA water and plankton (larvae) versus results from traditional sampling. Dreissenid mussel community compositions were compared between two allopatric ecosystems in Lake Erie and the Hudson River, which have separate invasion histories. Field experiments evaluated community differences across a temporal collection series in Lake Erie encompassing the reproductive season, and along a spatial gradient along the Hudson River. We also compared and contrasted species‐ and population‐genetic variability from two sites in Lake Erie, one in the western basin where the zebra mussel is more prevalent and the other in the central basin, which is dominated by the quagga mussel. These are statistically compared with results from the Hudson River sites, which also are dominated by the zebra mussel.

Our assay aimed to simultaneously assess inter‐ and intra‐specific genetic variation from up to thousands of individuals in environmental samples, allowing for detection from eDNA in water and plankton of early life stages. It was designed to alleviate morphological mis‐identifications. At the community level, accurate identification of dreissenids to species and assessment of their population genetic variability are projected to facilitate understanding their relative reproductive outputs, spread patterns, ecological interactions, successes, and adaptations over temporal and spatial scales.

## 2 MATERIALS AND METHODS

### 2.1 HTS assay design

First, we screened and aligned sequences for the “barcode” region of the mitochondrial (mt) cytochrome oxidase I (COI) gene from NIH NCBI GenBank (https://www.ncbi.nlm.nih.gov/genbank) and our own laboratory databases for all six species of *Dreissena *along with the two species of its sister genus *Mytilopsis* (Marshall & Stepien, 2019; Stepien et al., 2013). Primer pairs were designed by evaluating the aligned sequences with ClustalX in MEGA7 (Kumar, Stecher, & Tamura, 2016) and selecting regions of diagnostic variation that are flanked by conserved regions. The aligned database identified 20 unique zebra mussel haplotypes (labeled ZM–A through ZM–T) and 17 quagga mussel haplotypes (labeled QM–A through QM–Q) in a 570‐nucleotide (nt) region of COI (Supporting information Tables A1–A4). Two markers were developed and tested to target different nonoverlapping variable regions of ~200 nucleotides (nt), which encompassed intraspecific variation for zebra and quagga mussels and species‐specific diagnosis for all six species of *Dreissena, *along with the two species of the closely related genus* Mytilopsis *(see Stepien et al., 2013 for taxonomy). We then focused on testing the two markers on zebra and quagga mussels, which were designed to each distinguish different single nucleotide polymorphisms (SNPs).

Primer set 1 (COIA–F: 5'AGTGTTYTKATTCGTTTRGAGCTWAGKGC3‘, –R: 5'GAYAGGTARAACCCAAAAWCTWAC3’) flanks a 169‐nt region that contains 24 fixed differences between zebra and quagga mussels. It delineates 10 zebra mussel and eight quagga mussel haplotypes and differentiates among all six *Dreissena *species (Tables A1–A2). Primer set 2 (COIB–F: 5’GRAAWCTKGTMACACCAATAGAWGT3‘), –R: 5’GRAAWCTKGTMACACCAATAGAWGT3’) flanks a 175‐nt region with 26‐nts that differentiate zebra and quagga mussels. It delineates seven zebra mussel and nine quagga mussel haplotypes and also distinguishes among all six *Dreissena *species (Tables A3–A4). Our two primer sets distinguish seven of the nine quagga mussel COI haplotypes described by Marescaux et al. (2016), found throughout its native and invasive ranges.

### 2.2 Mock communities and aquaria experiments

Performance of the assays was tested on eight mock communities containing various known concentrations of DNA zebra and quagga mussel haplotypes (termed amplicon sequence variants (ASVs; see Callahan, McMurdie, & Holmes, 2017)). We thus tested whether the mock communities statistically maintained the relative ASV proportions during HTS (following our method previously published in Klymus, Marshall, & Stepien, 2017). Mixtures of purified DNA containing various proportions of 47–6,000 copies each of five zebra mussel haplotypes (ZM–A, ZM–C, ZM–G, ZM–M, and ZM–O) and three quagga mussel haplotypes (QM–A, QM–F, and QM–G) for the COI gene were analyzed (Table 1). We quantified the target copy number per DNA extraction for each ASV in a series of competitive polymerase chain reactions (PCR) that co‐amplified the native template (NT) and an internal standard (IS; see Klymus et al., 2017). Our COIA assay (Tables A1 and A2) was designed to differentiate among three zebra mussel haplotypes (ZM–C, ZM–G, and the ZM–A,M,O group) and three quagga mussel haplotypes (QM–A, QM–F, and QM–G; Tables A1 and A2); thus, six ASVs were identifiable in the mock community with this marker. The COIB assay was designed to differentiate four zebra mussel haplotypes (ZM–G, ZM–M, ZM–O, and the ZM–A,C group) and one quagga mussel haplotype group (QM–A,F,G group); thus, five ASVs would be diagnosable in the mock community (Tables A3 and A4).

**Table 1:** Sequence copy numbers of each species and haplotype that were used per mock community (MC 1–8), of the target mitochondrial DNA cytochrome oxidase I (COI) gene amplicon

Haplotype	Mock Communities							
	1	2	3	4	5	6	7	8
QM–A	6,000	3,000	1,500	750	375	188	94	47
ZM–A	3,000	1,500	750	375	188	94	47	6,000
ZM–M	1,500	750	375	188	94	47	6,000	3,000
ZM–C	750	375	188	94	47	6,000	3,000	1,500
QM–G	375	188	94	47	6,000	3,000	1,500	750
QM–F	188	94	47	6,000	3,000	1,500	750	375
ZM–G	94	47	6,000	3,000	1,500	750	375	188
ZM–O	47	6,000	3,000	1,500	750	375	188	94

Note

ZM = Dreissena* polymorpha* (zebra mussel), QM = *D. rostriformis* (quagga mussel). Haplotypes are lettered.

**Figure 2:**
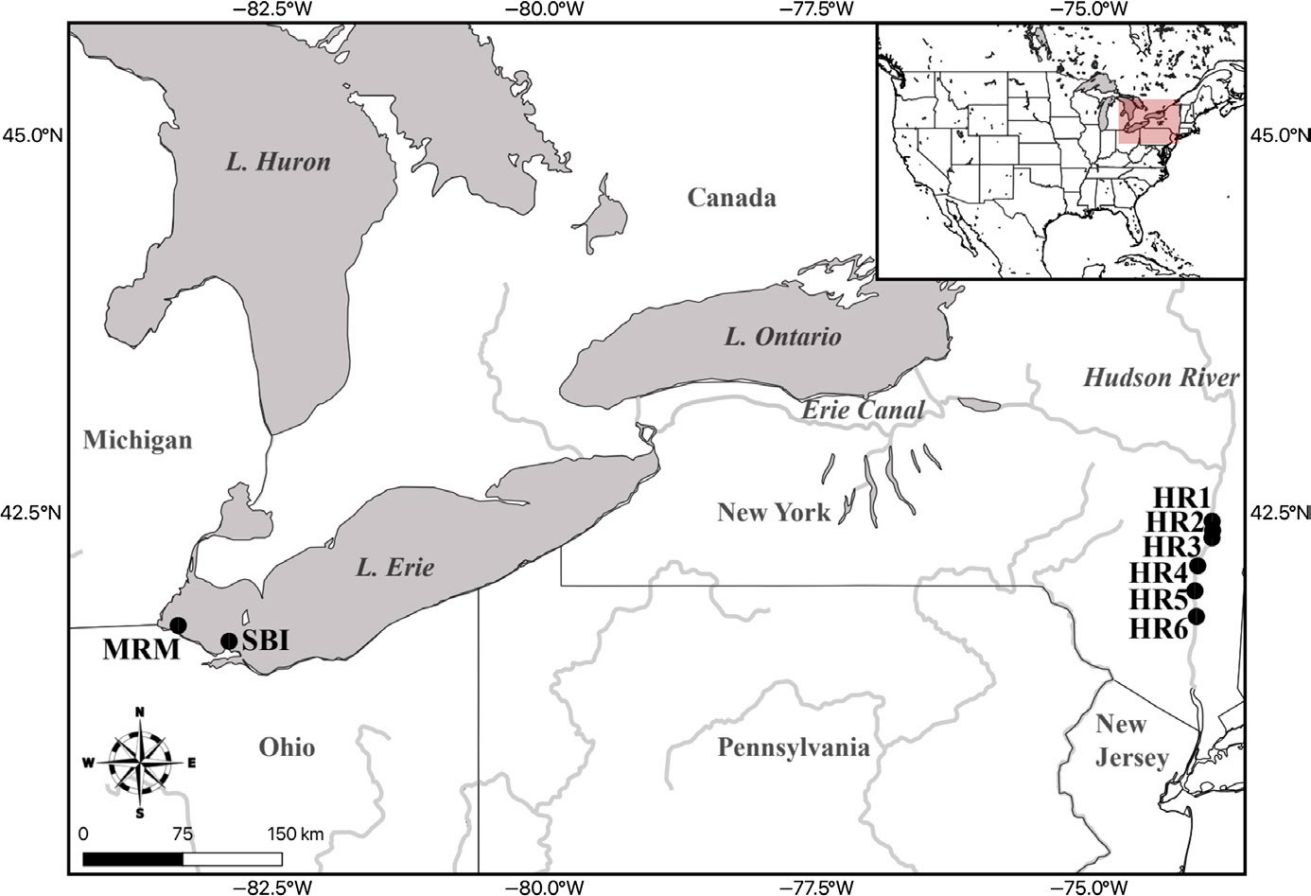
Site map for dreissenid mussel plankton and eDNA water samples, from the Hudson River (HR, locations 1–6) and Lake Erie (Maumee River's mouth (MRM) and South Bass Island (SBI))

Adult quagga mussels were collected from Belle Isle in the Detroit River, Detroit, MI (42.34, −82.99), and zebra mussels from the western Lake Erie basin in Oregon, OH (41.67, −83.29), both in April 2016 (Figure 2). Three 38‐L aquaria, air stones, and tubing were cleaned with 10% bleach and filled with 15 L of dechlorinated tap water. Aquaria were covered in clean plastic wrap and oxygenated throughout the experiments. Prior to introduction of the mussels, the water in each aquarium was thoroughly mixed, and a 500 ml aliquot was taken using a sterile container for eDNA analysis; this served as a negative control. The removed water was replaced with dechlorinated water from a sterile container. Mixed proportions of adults of the two species, ranging from 26 to 36 individuals total, were placed into each aquarium (Table 2). At days 2, 7, and 14, the water in each aquarium was sampled as above for eDNA analysis.

**Table 2:** Numbers *(N)*, weight (g), and percentage of species and haplotypes (lettered) used in aquarium environmental DNA experiments (Aquaria 1–3) with assay COIA. Mussels were weighed at the end of the experiment (at 14 days)

Species and Haplotype	Aquarium 1				Aquarium 2				Aquarium 3			
	*N*	%	Mass (g)	*%*	*N*	%	Mass (g)	%	*N*	%	Mass (g)	%
ZM–A	24	66.67	3.03	60.21	12	38.71	1.14	35.88	15	57.69	2.68	29.43
ZM–M	6	16.67	0.75	14.58	5	16.13	0.65	18.97	1	3.85	0.06	0.63
Total Zebra Mussels	30	83.33	3.78	74.79	17	54.84	1.79	51.80	16	61.54	2.74	30.06
QM–A	6	16.67	1.28	25.21	13	41.94	1.56	45.30	10	38.46	6.37	69.94
QM–G	0	0.00	0	0.00	1	3.23	0.10	2.90	0	0.00	0	0.00
Total Quagga Mussels	6	16.67	1.28	25.21	14	45.16	1.66	48.20	10	38.46	6.37	69.94
Total Mussels	36		5.06		31		3.45		26		9.11	

Water samples (including the negative controls) each were filtered through a 0.2‐µm polyethersulfone (PES) filter, from which eDNA was extracted using the Qiagen DNeasy^®^ Blood and Tissue Kit (Qiagen Inc., Valencia, CA, USA), with the amounts of proteinase *K* and ATL doubled in order to fully submerse the filter. Extracted DNA was run through a Zymo Research One Step PCR Inhibitor Removal kit (http://www.zymoresearch.com/rna/rna-clean-up/rt-pcr-inhibitor-removal/onestep-pcr-inhibitor-removal-kit).

At the end of the experiment (day 14) and after the final water collection, mussels were removed, air dried for one hour, and individually weighed (g). They then were slowly cooled in a refrigerator for 24 hr and then individually labeled and frozen. DNA from each was extracted, amplified, and Sanger sequenced for the COI gene using primers and methodology from Folmer, Black, Hoeh, Lutz, and Vrijenhoek (1994). PCRs contained 1X Radiant^®^ (Alkali Scientific) PCR buffer, 0.75 mM dNTPs, 3 mM MgCl_2_, 0.5 μM of each primer, 1.5U Radiant^®^ Taq, 100 ng of template DNA, and the necessary volume of ddH_2_0 to comprise 25 μl. The haplotypes were compared to those in GenBank and our laboratory database (Stepien et al., 2013).

### 2.3 Field eDNA water and plankton samples

Paired 1L water samples were taken for eDNA analysis from three Hudson River locations (Figure 2), including: Stuyvesant, NY (abbreviated HR1; 42.39, −73.78; depth = ~3.7 m), Coxsackie, NY (HR2; 42.35, −73.80; located four km downriver from Stuyvesant; depth= ~3.4 m), and Stockport, NY (HR3; 42.31, −73.77; located five river km downriver from Coxsackie; depth = ~4.3 m), on August 6, 2016. They were collected in sterilized bleach‐washed containers, one from 15 cm below the surface and the other 15 cm above the benthos (the latter were taken and capped by a team of SCUBA divers from the Cary Institute of Ecosystem Studies, who were careful not to touch or disturb the benthos). Water samples were labeled, placed on ice in the field, and then frozen at −20°C in the laboratory. Simultaneously, the SCUBA diving team surveyed the adult dreissenid mussel communities by collecting 10–12 rocks (15–40 cm in size), from which the mussels were counted in the Cary Institute laboratory (totaling 2,984 mussels from Stuyvesant, 2,174 from Coxsackie, and 3,345 from Stockport). Length–mass regression analysis was performed on a representative range of sizes for ~300 mussels to estimate respective biomass per species. In the laboratory, half of each water sample (500 ml) was pelleted in 10 sterile 50‐mL falcon tubes at 7,500 g centrifugation for 30 min. DNA was extracted with a cetyl‐trimethyl ammonium bromide (CTAB) protocol (Turner et al., 2014), and purified with a Zymo Research One Step PCR Inhibitor Removal kit, with the resultant DNA used for HTS.

Triplicate plankton samples were collected from four Hudson River sites (Figure 2)—Stuyvesant, NY (HR1), North Germantown, NY (HR4; 42.16, −73.89), Rhinecliff, NY (HR5; 41.92, −73.95), and Poughkeepsie, NY (HR6; 41.72, −73.94)—on May 31, 2016, with a 63‐µm Wisconsin Cole‐Parmer 40‐AD50 plankton net (https://www.coleparmer.com) attached to a 15.2 m line. The Poughkeepsie site was resampled on June 2, 2016, and August 6, 2016. For comparative analyses, plankton also was taken from two Lake Erie locations, at the Maumee River's mouth (MRM; 41.69, −83.43; ~1.5 m deep, sandy bottom, zebra mussel refugium) and South Bass Island (SBI; 41.63, −82.84; ~5 m deep, rocky bottom, quagga mussel dominated), in the afternoon every two weeks from June through September 2016. The net was thrown from the seawall/shore and, upon sinking, was maintained at the maximum depth the line would allow for 60 s, then manually retrieved using a hand‐over‐hand technique ~0.5 m/s, resulting in ~0.19 m^3^ (~190 L) of water sampled. Upon retrieval, each sample was placed into labeled 15‐mL falcon tubes containing 95% EtOH.

Numbers of *Dreissena *veliger larvae per site from the plankton tows were estimated from three separate 500 µl aliquots using a Sedgewick‐Rafter counting cell (Thermofisher^®^, https://www.thermofisher.com) under cross‐polarized light microscopy, following Johnson (1995). Density (number of *Dreissena *veligers per liter; Hosler, 2011) was determined from the mean of the three aliquots per sample, multiplying by the sample volume (15 ml) divided by the filtered volume (~190 L), and reported as the mean of replicates ± standard error (*SE*). Numbers of *Dreissena *larvae were statistically compared using paired Student's *t* tests (Sokal & Rohlf, 1994). One plankton sample from each site was centrifuged at 6,000 rpm for 5 min, and the ethanol decanted. DNA was extracted from the resultant pellet using the CTAB soil extraction protocol (Turner et al., 2014), with the resultant DNA amplified for HTS.

### 2.4 MiSeq HTS library preparation

A four‐step PCR library preparation was used prior to paired‐end HTS on the Illumina^®^ MiSeq platform (https://www.illumina.com/systems/sequencing-platforms/miseq.html). After each PCR, products were column cleaned with QIAquick^®^ PCR purification kits (Qiagen; https://www.qiagen.com), yielding the template for the subsequent step. The first step of four cycles used assay‐specific primers (COIA or COIB) to append 21‐nt APEX tails (F:5’GATCAGGCGTCTGTCGTGCTC3’, R:5’CTCGACGACAGACGCCTGATC3’) to the target regions, which aimed to reduce PCR bias by retaining the relative ASV proportions of the sample throughout library preparation (Krjutškov et al., 2008). PCRs contained 1X PCR buffer, 0.2 mM dNTPs, 0.5 μM of each primer, 1U AmpliTaq^®^, 2.5 μl template DNA, and the necessary volume of ddH_2_0 to constitute 25 μl. Conditions were as follows: 30‐s initial denaturation at 95°C, followed by four cycles of 95°C for 30‐s, 62°C for 30‐s, and 72°C for 1‐min, with 2‐min final extension at 72°C. Next, the APEX primers were used to amplify targets adhered with the APEX tail, using the same PCR conditions for 35 cycles.

The third step amplified previous products using APEX primers appended with 7‐ to 17‐nt (termed spacers) and a 33‐ to 34‐nt sequencing primer on the 5’ end. The spacer region increased nt diversity of sequence reads and enhanced cluster formation, improving sequencing quality (Fadrosh et al., 2014; Wu et al., 2015). Four primer sets were used, which differed in their spacer combinations (see Klymus et al., 2017). PCR conditions were identical, except that the cycle number was reduced to eight. The final (fourth) PCR incorporated Nextera paired‐end indices (Illumina^®^, kit FC‐121‐1011), as well as the P5 and P7 adaptor sequences, allowing the sample to bind onto the Illumina^®^ MiSeq flowcell. The 25 μl PCRs contained a variable volume of ddH_2_0, 1× PCR buffer, 0.2 mM dNTPs, 2.5 μl of each primer, 1.57U NEB Hotstart Taq polymerase (New England Biolabs^®^, Inc.), and 2.5 μL of the previous PCR cleanup. Conditions were as follows: 30‐s initial denaturation at 95°C, followed by eight cycles at 95°C for 30‐s, 55°C for 30‐s, and 68°C for 1‐min, with a final 2‐min 68°C extension. Four samples were run with the Mol16S primer set followed the library preparation, which was published in Klymus et al. (2017).

To avoid sequencing dimer product, targeted fragments first were size‐selected with a 1.5% agarose gel cassette on Pippin Prep (Sage Science). PCR products were sized and quantified on a 2100 Bioanalyzer (Agilent Technologies). A negative control was run for each library preparation step and checked for contamination with gel electrophoresis, with the final step checked on the 2100 Bioanalyzer to verify the absence of product. No contamination was found in any instance. Pooled product concentrations were measured on a Qubit fluorometer (Invitrogen), and the pooled samples then were run on an Illumina^®^ MiSeq with 2X 300nt V3 chemistry at the Ohio State University's Molecular and Cellular Imaging Center in Wooster, OH (http://mcic.osu.edu/). An additional 40%–50% PhiX DNA spike‐in control was added to improve data quality of low nt diversity samples (Klymus et al., 2017). HTS was performed on four MiSeq runs, which also contained tagged samples from other projects to increase diversity; all were pooled to yield >100,000 reads per sample.

### 2.5 Bioinformatic and statistical analyses

MiSeq results were analyzed using the *R *package DADA2 (Callahan et al., 2016), except for three mock community samples that had low read depths (COIA mock community 5, and COIB mock communities 2 and 7). We instead merged and trimmed those with the Python package OBITools 1.2.11 (Boyer et al., 2016). For the other samples, the primer and spacer regions first were trimmed using a custom Perl script. Next, using the default settings in DADA2, error rates were estimated, sequences were merged and grouped into ASVs, and any erroneous sequences and chimeras were removed. Chimeras were not removed from the COIB dataset, since DADA2 mistakenly regarded haplotype ZM–E as a chimeric sequence. Sequence ASVs were identified to dreissenid species and haplotypes in reference to COI sequences on GenBank and our own database. Possible erroneous ASVs were eliminated from our environmental datasets by removing any sequence present only in a single sample.

For mock communities, the percentage of reads per ASV was calculated and analyzed using *R *v3.4.3 (R Core Team, 2015) by regressing the logarithm of their observed versus expected percent read abundances to evaluate goodness of fit and whether the slopes differed from 1.00. Contingency tests (Sokal & Rohlf, 1994) were used to compare HTS reads from (a) aquarium eDNA water having known ASV compositions, (b) Hudson River eDNA water samples with traditional morphological census counts, and (c) plankton across temporal and spatial scales. To compare relatedness among multiple‐ASV community assemblages, Nonmetric multidimensional scaling (NMDS; Kruskal, 1964) ordinations were performed using Bray–Curtis dissimilarity indices of the relative abundances of ASVs using the *R* package vegan (Oksanen et al., 2018) and the *metaMDS* and *anosim *functions to parse the data according to collection locations. Differences in the relative abundances of ASVs among the samples were evaluated using the pairwise Kulczynski (1927) Dissimilarity Index (KDI), which ranged from 0 (equal) to 1 (completely different).

## 3 RESULTS

### 3.1 HTS of mock communities and aquaria

**Figure 3:**
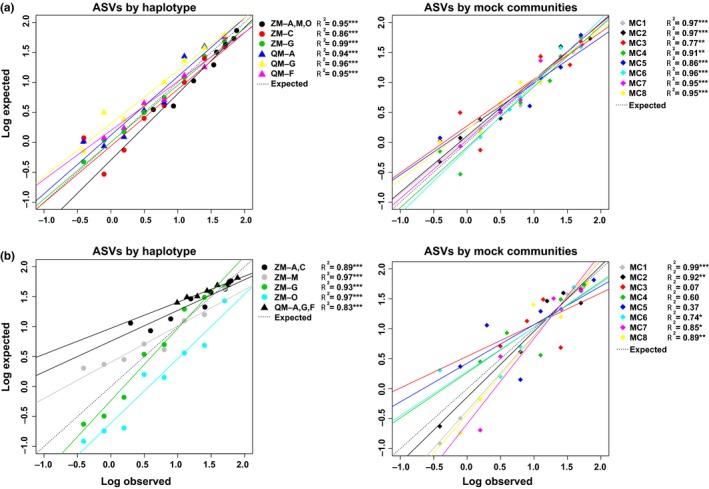
Regression analyses of log transformed observed versus expected read percentages per haplotype (lettered and colored) of zebra mussel (ZM) and quagga mussel (QM) and mock community (MC) for the two assays (a) COIA and (b) COIB. Significant at * = 0.05, ** = 0.01, *** = 0.001

Number of HTS reads passing cluster quality filtering ranged from 14,329,380 to 15,237,283 per run (Table A5). Regression analyses revealed significant matches (*p* < 0.05) between the observed and expected sequence read percentages for the mock community samples with the COIA primers (Figure 3a; Table 3), corresponding to the expected slope = 1.00. In contrast, the COIB marker displayed a weaker relationship between observed and expected ASV proportions for mock communities 3–5 (*p* > 0.05; Figure 3b; Table 3), with the slopes of 3–7 significantly differing from 1.00 (ranging from 0.53 to 1.42). As the COIB marker yielded inconsistent results with the mock communities, it was not used for further analysis with the aquaria or environmental samples. However, the HTS results between the two markers did not significantly differ for determining overall species composition in six of the eight mock communities, and both showed similar trends between observed and expected ASV proportions.

**Table 3:** Numbers of sequence reads and observed and expected percentages per taxon and mock community (MC 1–8) for the two assays (COIA and COIB)

Assay	Haplotype	MC 1			MC 2			MC 3			MC 4		
		Read number	% Obs.	% Exp.	Read number	% Obs.	% Exp.	Read number	% Obs.	% Exp.	Read number	% Obs.	% Exp.
COIA	ZM–A,M,O	44,985	31.4	38.0	99,521	53.7	69.0	42,468	20.6	34.5	21,288	10.9	17.3
	ZM–C	5,649	3.9	6.3	5,058	2.7	3.1	1687	0.8	1.6	602	0.3	0.8
	ZM–G	1695	1.2	0.8	1544	0.8	0.4	98,326	47.8	50.2	52,667	26.9	25.1
	QM–A	82,114	57.3	50.2	71,510	38.6	25.1	54,804	26.6	12.6	8,828	4.5	6.3
	QM–G	6,321	4.4	3.1	4,521	2.4	1.6	6,454	3.1	0.8	1540	0.8	0.4
	QM–F	2,577	1.8	1.6	3,237	1.7	0.8	2024	1.0	0.4	111,060	56.7	50.2
COIB	ZM–A,C	125,618	37.8	31.4	681	28.8	15.7	34,876	14.2	7.9	17,288	9.1	3.9
	ZM–M	43,243	13.0	12.6	66	2.8	6.3	13,122	5.3	3.1	5,523	2.9	1.6
	ZM–G	1,040	0.3	0.8	5	0.2	0.4	114,816	46.6	50.2	61,521	32.4	25.1
	ZM–O	508	0.2	0.4	641	27.1	50.2	12,560	5.1	25.1	7,076	3.7	12.6
	QM–A,F,G	161,660	48.7	54.9	974	41.1	27.5	70,771	28.8	13.7	105,464	55.6	56.9

Assay	Haplotype	MC 5			MC 6			MC 7			MC 8		
		Read number	% Obs.	% Exp.	Read number	% Obs.	% Exp.	Read number	% Obs.	% Exp.	Read number	% Obs.	% Exp.
COIA	ZM–A,M,O	58	4.0	8.6	9,389	3.7	4.3	42,379	43.2	52.2	94,049	72.5	76.1
	ZM–C	17	1.2	0.4	97,801	38.8	50.2	24,253	24.7	25.1	13,038	10.1	12.6
	ZM–G	173	12.0	12.6	14,465	5.7	6.3	3,045	3.1	3.1	2,342	1.8	1.6
	QM–A	49	3.4	3.1	3,152	1.2	1.6	906	0.9	0.8	1,264	1.0	0.4
	QM–G	886	61.5	50.2	94,738	37.6	25.1	22,516	23.0	12.6	12,859	9.9	6.3
	QM–F	257	17.9	25.1	32,723	13.0	12.6	4,911	5.0	6.3	6,110	4.7	3.1
COIB	ZM–A,C	30,616	12.7	2.0	143,328	43.3	51.0	539	21.8	25.5	180,808	59.3	62.7
	ZM–M	6,448	2.7	0.8	7,192	2.2	0.4	1,066	43.1	50.2	53,123	17.4	25.1
	ZM–G	49,714	20.6	12.6	18,299	5.5	6.3	78	3.2	3.1	2082	0.7	1.6
	ZM–O	4,003	1.7	6.3	5,842	1.8	3.1	5	0.4	1.6	747	0.2	0.8
	QM–A,F,G	154,937	64.1	78.4	162,485	49	39.2	784	31.7	19.6	68,943	22.6	9.8

Note

*Z*M = Dreissena* polymorpha* (zebra mussel), QM = *D. rostriformis* (quagga mussel). Haplotypes are lettered.

**Figure 4:**
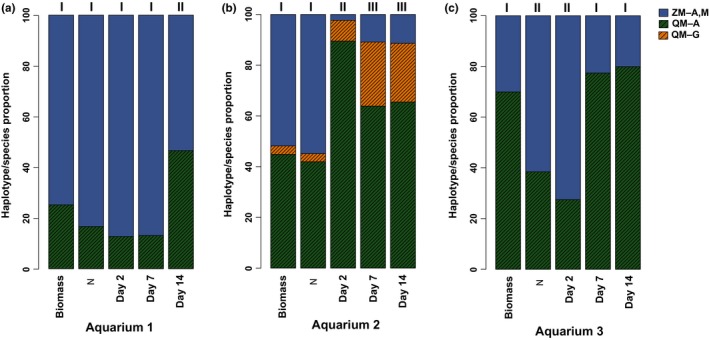
Species and haplotype proportions of zebra mussels (ZM; *Dreissena polymorpha*) and quagga mussels (QM; *D. rostriformis*) from environmental DNA in three aquarium experiments (A–C) using our targeted high‐throughput sequencing COIA assay, in relation to biomass (weight, g) and number of individuals (N). Water samples taken on days 2, 7, and 14. Haplotypes are lettered and colored. Roman numerals denote significant concordance and/or differences in relative proportion of sequence reads for each aquarium using contingency *G*‐tests

None of the negative controls from the three aquaria amplified, indicating that the water, aquaria, and aeration systems were free of DNA contamination. Both species and all of their expected haplotypes were identified with HTS from each aquarium on all dates (Figure 4). Biomass of the dreissenids significantly differed among all aquaria (***p = 0.001), whereas the numbers of individuals per species significantly differed just between aquarium #1 and the other two (#1 *vs*. 2, #1 *vs*. 3: ***p < 0.001). Numbers of individuals per species versus their relative biomass significantly differed for #3 alone (***p < 0.001, Figure 4). For #3, the zebra mussels were relatively small in size in comparison with the quagga mussels; thus, the relative biomass of the latter was much greater than their relative numbers (Table 2).

Species proportions in the HTS reads from aquarium #1 at days 2 and 7 did not significantly differ from the relative numbers of individuals per species or from biomass (Figure 4a). On day 14, aquarium #1's sequence reads significantly differed from both its numbers of individuals per species (***p < 0.001) and biomass (***p < 0.001). Sequence reads from aquarium #2 significantly differed from numbers of individuals per species and biomass on all three days (Figure 4b). A quagga mussel died in aquarium #2, which went unnoticed. It likely died early in the experiment, increasing the amount of quagga mussel DNA throughout the duration. Sequence reads per species in aquarium #3 did not differ from the number of individuals per species at day 2, but differed from their respective biomass (***p < 0.001; Figure 4c). However, at days 7 and 14, its sequence reads did not differ from biomass per species, but varied from the numbers of individuals per species (***p < 0.001). This result resembled that of aquarium #1.

By the end of the experiment, the relative proportions of all sequence reads per species significantly differed from those at day 2 (day 2 vs. 14: ***p < 0.001 for all three aquaria; Figure 4), revealing a trend that more closely matched biomass at day 14, rather than the numbers of individuals (as occurred on day 2). The change to resembling biomass per species was observed at day 7 for aquaria #2 and #3, but at day 14 in #1.

### 3.2 HTS of field eDNA water and plankton samples

**Figure 5:**
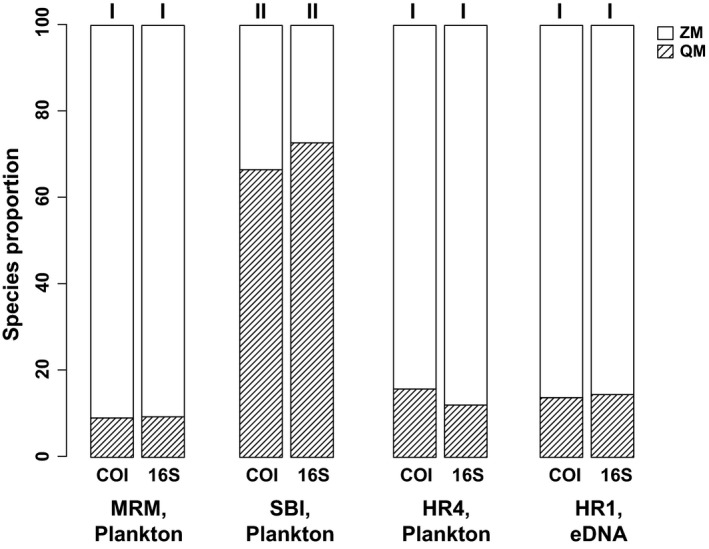
Species proportions of zebra mussels (ZM; *Dreissena polymorpha*) and quagga mussels (QM; *D. rostriformis*) from plankton and eDNA field experiments using our targeted high‐throughput sequencing COIA assay (this paper) and our previously published 16S assay (Klymus et al., 2017). Sampling locations were as follows: Maumee River's mouth (MRM) in Lake Erie (July 1, 2016), South Bass Island (SBI) in Lake Erie (July 1, 2016) and Hudson River (HR4, at North Germanton, NY on May 31, 2016, and HR1 at Stuyvesant, NY, on August 6, 2016). Roman numerals designate concordance among samples at sites using contingency *G*‐tests

The proportions of sequence reads obtained per species did not statistically differ between the new COIA (COI) assay (this study) and results using our previously published mollusk mtDNA 16S RNA (16S) assay (from Klymus et al., 2017), for the three plankton collection locations (ZM:QM proportions at the Maumee River's mouth (MRM), Lake Erie: 16S–9:91% versus COI–9:91%; South Bass Island (SBI), Lake Erie: 16S–73:27% versus COI–67:33%; North Germantown, Hudson River (HR4):16S–16:84% versus COI–12:88%; Figure 5). The read proportions from each assay also did not statistically differ in the eDNA water sample (Stuyvesant, Hudson River (HR3): 16S–14:86% versus COI–15:85%; Figure 5). The COIA was dreissenid specific, as 100% of the HTS results corresponded to zebra and quagga mussels. In contrast, the proportion of dreissenid sequences was much lower for the 16S assay (ranging from 0.36% to 80.25%).

**Figure 6:**
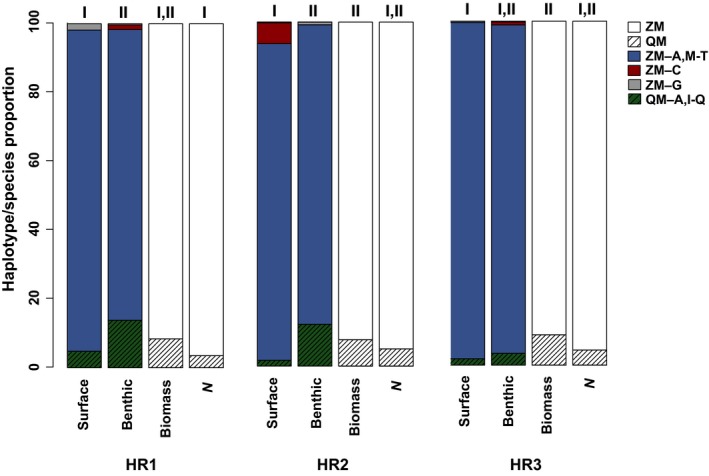
Species and haplotype proportions of zebra mussels (ZM; *Dreissena polymorpha*) and quagga mussels (QM; *D. rostriformis*) from field eDNA samples using our targeted high‐throughput sequencing COIA assay, in relation to estimated biomass (weight, g) and number of individuals (*n*) in the adult community of the Hudson River. eDNA water samples collected from the surface and the benthos at three sites (Stuyvesant, NY (HR1), Coxsackie, NY (HR2), and Stockport, NY (HR3)). Haplotypes are lettered and colored. Roman numerals denote significant concordance and/or differences in relative proportion of sequence reads per site using contingency *G*‐tests

Additionally, the proportions of species‐specific sequence reads from the Hudson River eDNA water samples were statistically similar to the species ratios of the benthic adults determined by the Cary Institute's traditional morphological identifications (Figure 6). In both data sets and at all locations, zebra mussels greatly outnumbered quagga mussels. The Cary Institute's proportions were 95.0%–96.5% (mean = 95.7%) zebra mussels from morphological counts and 91.2%–92.3% (mean = 91.7%) zebra mussels using estimated biomass. Their species’ proportions did not significantly differ among the three Hudson River locations. Our HTS reads from eDNA water (surface and benthic samples) similarly yielded 83.5%–97.7% (mean = 92.1%) zebra mussels. Total read proportions (surface + benthic eDNA) did not significantly differ among the locations. Overall, the average ZM:QM proportions (surface + benthic eDNA) did not significantly differ between the HTS results and the Cary Institute's morphological counts or from the estimated biomass at any site.

For the eDNA water collected near the benthos, the HTS species’ proportions differed between Stuyvesant versus Stockport (**p = 0.006) and Coxsackie versus Stockport (*p = 0.01), whereas none of the three surface samples differed. Species proportions significantly varied between the surface and benthic eDNA at Stuyvesant (*p = 0.02) and Coxsackie (**p = 0.001). The HTS species proportions (93.9%–97.7%, mean = 96.5%) from the surface eDNA water more closely matched the Cary Institute's morphological species counts (no significant differences at any site) than did their biomass estimates (significant variations at Stockport, *p = 0.02 and Coxsackie, *p = 0.04; Figure 6). The benthic eDNA sequence reads (83.5%–95.5% zebra mussels, mean = 87.8%) more closely matched the Cary Institute's biomass estimates (no significant differences at any site), than their adult mussel counts (significant difference for Stuyvesant, *p = 0.02; Figure 6).

**Figure 7:**
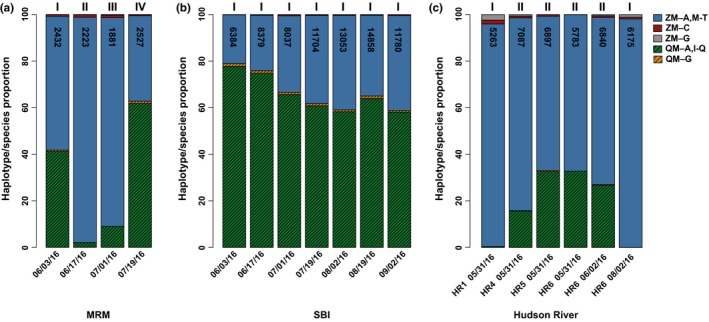
Species and haplotype proportions of zebra mussels (ZM; *Dreissena polymorpha*) and quagga mussels (QM; *D. rostriformis*) from field plankton samples collected from (a) Maumee River's mouth (MRM) in Lake Erie, (b) South Bass Island (SBI) in Lake Erie, and (c) the Hudson River at Stuyvesant, NY (HR1), North Germantown, NY (HR4), Rhinecliff, NY (HR5), and Poughkeepsie, NY (HR6), using our targeted high‐throughput sequencing COIA assay. Haplotypes are lettered and colored. Roman numerals denote significant concordance and/or differences in relative proportion of sequence reads for each location using contingency *G*‐tests. Numbers in columns are comparative dreissenid veliger larvae count estimates from plankton tows (three replicates each, counted under cross‐polarized light microscope)

Physical counts of *Dreissena* veliger larvae made under the microscope from the plankton samples were consistently lower from the Maumee River's mouth than at the South Bass Island and Hudson River locations (mean MRM = 11.9/L (2,261/sample); SBI = 55.8/L (10,602); HR = 35.7/L (6,783/sample)). The numbers of veligers significantly differed between the Lake Erie sites (Maumee River's mouth and South Bass Island) throughout the sampling season (***p < 0.001; Figure 7a,b). The relative HTS proportions of zebra mussels to quagga mussels (ZM:QM) in the plankton differed spatially across the Hudson River locations, revealing increasing proportions of quagga mussel veligers from upstream (north) to downstream (south; 0%–35%, mean = 20%). These species proportions significantly differed between the northern site at Stuyvesant (100% ZM) and the southern location at Poughkeepsie (70:30% ZM:QM, ***p < 0.001). Two plankton samples collected three days apart at Poughkeepsie yielded similar species proportions (64:36%, 67:33%), indicating short‐term temporal consistency, whereas the August 2, 2016, sample significantly differed from the earlier ones (***p < 0.001; Figure 7c).

In western Lake Erie, the relative HTS proportions of ZM:QM in plankton averaged 70:30% (ranging from 37%–97% ZM) at the Maumee River's mouth and 34:66% (22%–41% ZM) at South Bass Island, which differed significantly in depth (with the Maumee River's mouth being shallower) and were located ~36 km apart. Overall HTS proportions of ZM:QM significantly differed between the two locations (***p < 0.001), matching their adult communities. The species proportions significantly differed between the two sites on all common dates (June 3, ***p < 0.001; June 17, ***p < 0.001; July 1, ***p < 0.001), except July 19.

Species composition of the veliger larvae significantly varied temporally at the Maumee River's mouth (mean **p = 0.008), with fewer zebra mussels and more quagga mussels at the earliest (ZM:QM 57:43% June 3, 2016) and later dates (37:63% July 19, 2016). Zebra mussel veligers were overwhelmingly prevalent at the two intermediate dates (97:03% June 17; 90:10% July 1; Figure 7a). Dreissenid veligers were absent in the August and September microscope counts of the Maumee River's mouth samples, and DNA extractions from the plankton did not amplify. No significant temporal differences occurred in the HTS species proportions of ZM:QM larvae at the South Bass Island location, with quagga mussels always being more prevalent (Figure 7b).

**Figure 8:**
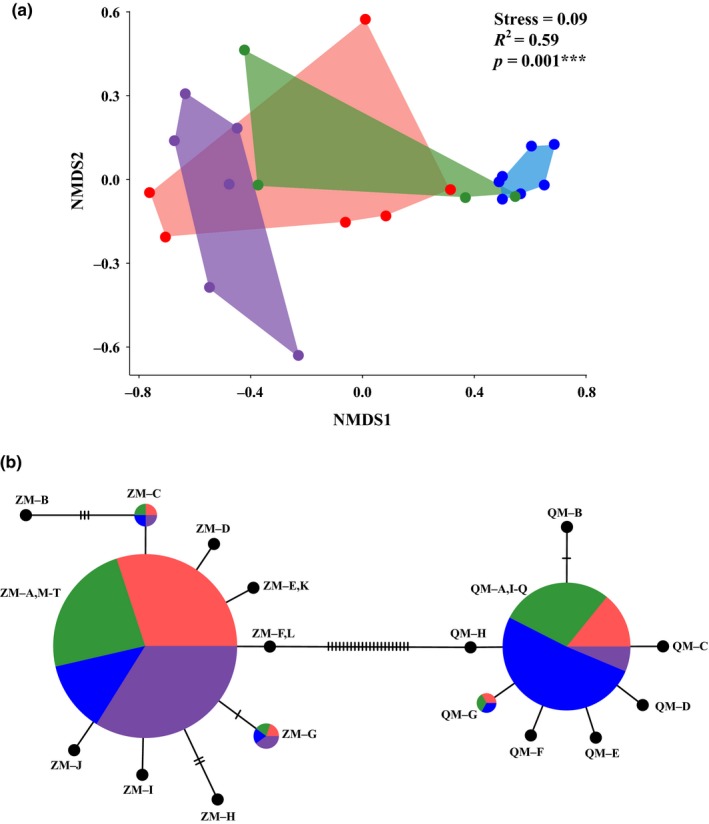
(a). Nonmetric multidimensional scaling (NMDS) plot (Bray–Curtis distance metric of relative abundance) showing the relationships among dreissenid mussel communities from field samples using our targeted high‐throughput sequencing COIA assay. Purple = Hudson River eDNA water, Red = Hudson River plankton, Green = Maumee River's mouth, Lake Erie plankton, Blue = South Bass Island, Lake Erie plankton. (b) Network showing relationships among zebra mussel (ZM; *Dreissena polymorpha*) and quagga mussel (QM; *D. rostriformis*) haplotypes for the mtDNA COI gene region used for our COIA assay. Hatch marks designate the number of nucleotide differences. Circle sizes represent the proportion of sequence reads attributed to that haplotype per sample location. Black circles denote haplotypes from NIH NCBI GenBank that were not found in our environmental samples

HTS species compositions of the larvae, averaged across all sampling dates per site, did not significantly differ between the Hudson River (all four locations added together) and the Maumee River's mouth, reflecting the common overall prevalence of zebra mussels at both. Samples of larvae from both locations significantly differed from South Bass Island, where quagga mussels dominated (MRM *vs*.SBI: ***p < 0.001; HR *vs*.SBI: ***p < 0.001). NMDS likewise depicted the South Bass Island larval community as distinct from the other two (MRM *vs*.SBI: *p = 0.02; HR vs. SBI: ***p < 0.001; HReDNA vs. SBI: **p = 0.002; Figure 8a). Pairwise KDI tests supported the NMDS findings, with the lowest KDI values for each plankton sample typically corresponding to the others from the same location (Table A6). When samples were grouped by location, the lowest KDI values occurred between the Hudson River and Maumee River's mouth (KDI = 0.10), and the largest between the Hudson River versus South Bass Island (KDI = 0.58; Table A6).

### 3.3 Haplotypic diversity

Two rare zebra mussel haplotypes (ZM–C and ZM–G) and one rare quagga mussel haplotype (QM–G) were detected in the HTS analyses of eDNA water and plankton, with all three haplotypes previously known in GenBank. Haplotype ZM–C was present in all but one Lake Erie plankton sample on August 2 (0.87% at MRM and 0.33% at SBI) and occurred throughout all Hudson River sites (0.78% in plankton and 1.37% in eDNA; Figures 7 and 8b). We found the rare haplotype ZM–G throughout the Hudson River (four sites at 0.76% in plankton and all locations at 0.65% in eDNA), which was scarce in Lake Erie (one MRM sample at 0.07% and four from SBI at 0.04%). The QM–G haplotype was discerned in plankton at both Lake Erie locations (0.39% at MRM and 0.98% at SBI) and in the Hudson River (0.18%), but was absent from our Hudson River eDNA samples (Figure 8b).

## 4 DISCUSSION

This study developed and tested a new HTS metabarcoding assay that simultaneously differentiated among dreissenid species and their intraspecific haplotypes from single samples containing DNA from thousands of individuals. Since a single dreissenid mussel adult can release >1,000,000 gametes in a spawning season (Borcherding, 1991), and subsequent larval stages persist throughout the water column from early May through September (Borcherding, 1991; Sprung, 1993), water and plankton provide an opportunity to rapidly survey DNA from vast numbers of individuals, facilitating early and accurate detection of AIS. Estimated numbers of veliger larvae analyzed per our single plankton samples ranged from 2,220 to 14,860 individuals, demonstrating the utility of our assay and approach to assess the genetic variation of thousands of individuals. Thus, our metabarcoding approach offers greater ability to effectively discern species and population genetic diversity versus time‐intensive, single individual processing with traditional sampling and sequencing. This HTS assay also accurately reveals species proportions within varying environmental samples (both water and plankton), as our sequencing results matched those estimated using our laboratory's previously published mollusk 16S assay (Klymus et al., 2017). That 16S assay, unlike our new COI assay, does not resolve intraspecific variation. Furthermore, the 16S assay amplifies a wide range of taxa and thus might fail to detect a small dreissenid population.

The COI HTS assay was successful for evaluating representative haplotypic proportions of zebra and quagga mussels in laboratory and field tests. Sequencing results for the laboratory mock communities demonstrated strong relationship between observed and expected proportions. Aquaria experiments housing differing species compositions yielded results similar to their known species’ biomass proportions. Although the HTS results from the aquaria initially resembled the numbers of individuals per species at day 2, sequences from all three tanks best reflected biomass by day 14. This relationship may have lagged while the mussels acclimated to the aquaria by adjusting their filtration rates. Considering that the sequence read output per species more closely matched biomass after longer acclimation times (days 7 and 14), eDNA sampled from field collections also should accurately reflect the respective biomass of species compositions, illustrating widespread ecological application of our assay. Experiments with fishes (Takahara, Minamoto, Yamanaka, Doi, & Kawabata, 2012) and amphibians (Evans et al., 2016; Thomsen et al., 2012) likewise found that eDNA quantification from mesocosms correlated well with biomass of the target organisms.

Our HTS assay accurately characterized dreissenid mussel community‐level species compositions, along with their population genetic variability, from field‐collected water. HTS proportions from their eDNA revealed similar compositions to the adult dreissenid mussel communities, matching morphological counts and estimated biomass. In the Hudson River, HTS species determinations for the surface water closely resembled the morphological identification counts of the benthic adults, whereas the benthic water showed greater quagga mussel proportions, which more closely resembled the biomass estimates. Similarly, by day 14 of the aquaria experiments, quagga mussel HTS proportions were slightly greater than expected in all three aquaria, due to their larger biomass. This trend might be attributed to the larger sizes of the quagga mussel individuals, and possible physiological differences between the species. For example, a study by Diggins (2001) found that quagga mussels had higher filtration rates (>37%) than zebra mussels of the same size (20 mm), which could lead to higher rates of eDNA shedding, but remains to be experimentally verified. In our study, when the surface and benthic eDNA results were averaged together, their species’ proportional sequence reads did not differ from either their morphological counts or estimated biomass at any field location. These results suggest that slight differences in DNA concentrations can occur throughout the water column, and water collected from near the benthos (where most adults live) would best represent the true dreissenid biomass composition. A qPCR study of zebra mussel eDNA also found correspondence to benthic biomass of adults (Amberg, Merkes, Stott, Rees, & Erickson, 2019). As in the present study, eDNA surveys in the Rhine River using species‐specific quantitative (q)PCR assays matched the respective species proportions from field surveys (De Ventura et al., 2017). Their approach differed from ours as they required two different probes to differentiate between the species and were unable to evaluate haplotypic (intraspecific) variation.

Sequencing results with our HTS assay accurately depicted the two allopatric dreissenid invasions, with the Hudson River and South Bass Island in Lake Erie sites being the most divergent. These results matched the morphological species compositions of the two invasions, with the quagga mussel dominating at South Bass Island (Karatayev et al., 2014) and the zebra mussel most prevalent in the Hudson River (Strayer & Malcom, 2013). As expected, our assay showed that the zebra mussel dominated the plankton from the Maumee River's mouth, since that region has been described as having the sole zebra mussel refugium in Lake Erie (Karatayev et al., 2014). Additionally, our assay displayed temporal resolution at the Maumee River's mouth, whose larval species composition varied throughout the sampling season. Throughout June, its species composition more closely resembled that from the Hudson River, but then changed to resemble South Bass Island on the final sampling date (July 19). This may have been due to larval transport, as Lake Erie experienced strong westward winds during that time period (wind data from the NOAA National Data Buoy Center (NDBC) at buoy SBI01 located at South Bass Island, and buoy THR01 at the Maumee River's mouth; http://www.ndbc.noaa.gov). These winds may have caused an influx of quagga mussel veliger larvae traveling from central Lake Erie to the west. For the Hudson River, HTS of eDNA water and plankton agreed in species composition and haplotypic diversity representation, indicating that both sampling techniques yield similar results. These complementary collection methods thus surveyed a large breadth of dreissenid inter‐ and intra‐specific variation in situ.

The quagga mussel has been outcompeting the zebra mussel in many invaded European rivers, increasing by an estimated 26% per year in Germany and the Netherlands (Heiler et al., 2013). Large fluctuations in their densities, with quagga mussels eventually displacing zebra mussels, were observed in the northern Dnieper River (Ukraine; Zhulidov et al., 2010). A similar takeover by the quagga mussel might substantially alter ecological processes in the Hudson River. For example, the quagga mussel's displacement of the zebra mussel in the lower Great Lakes increased total dreissenid biomass, as the quagga mussel more readily spreads across soft substrates, magnifying overall ecological impacts (Nalepa, Fanslow, & Lang, 2009). Moreover, selective filtering and phosphorous excretion appear to be contributing to increased harmful algal blooms in the lower Great Lakes and may significantly differ between the two dreissenid species (Conroy et al., 2005; Vanderploeg et al., 2001). Therefore, it is important to effectively discern the species compositions of dreissenid communities spatially and temporally. This can be effectively accomplished through analysis of their abundant planktonic larvae, which were previously indistinguishable at the species and population levels. Thus, our assay can facilitate effective and accurate detection, and monitoring, of dreissenid communities via the simultaneous metabarcoding of thousands of veligers from plankton.

Although our assay solely was used here for zebra and quagga mussel populations, it could further be applied to distinguish the presence and species identities of the four other *Dreissena *species, as well as the two related—and also highly invasive—*Mytilopsis *species (*M. leucophaeata *and *M. sallei*). In marine and estuarine harbors worldwide, invasive *Mytilopsis* spp. exert similar ecological and economic fouling problems as do zebra and quagga mussels (Laine, Mattila, & Lehikoinen, 2006; Sousa, Gutiérrez, & Aldridge, 2009). In addition to the well‐known zebra and quagga mussels, two *Dreissena* species native to the Balkans (*D. blanci *and *D. carinata*) recently expanded their ranges and may become invasive throughout Europe (Wilke et al., 2010). Furthermore, *D. anatolica *is endemic to just a few lakes in Turkey (Marshall & Stepien, 2019; Stepien et al., 2013; van der Velde et al., 2010) and therefore is at risk from competition pressures by future expansions of congeneric invaders. Our assay thus has apparent wide application to track and monitor the distributional trajectories of these taxa, by providing both species identification and their respective intraspecific population data.

Due to the much larger numbers of individuals surveyed (thousands vs. ~100), rarer haplotypes are more likely to be analyzed using HTS than with conventional sampling and sequencing of separate individuals. In our results, the rare haplotypes identified from surface and benthic collections at the same location sometimes differed; we thus recommend that future eDNA studies survey a variety of depths, with triplicate samples, to facilitate thorough evaluation of haplotypic diversity. Samples taken from different depths could be combined during DNA extraction, thereby yielding an enhanced representation of the entire water column, increasing the likelihood of surveying rare haplotypes. Site occupancy models also could be employed to evaluate possible failure to detect rare haplotypic variants (Dorazio & Erickson, 2017).

All haplotypes identified here for the zebra and quagga mussels are previously known sequences deposited in GenBank. This produces strong evidence that our screening procedure successfully removed any erroneous haplotypes resulting from possible PCR/sequencing error. Haplotype ZM–C previously was identified only in the Mohawk River, NY, a tributary of the Hudson River (Molloy et al., 2010), but here occurred in all of our Hudson River sites (plankton and eDNA), as well as in all but one of our Lake Erie plankton samples. ZM–G previously was described from the Dnieper River (a native habitat; Soroka, 2010) and Lake Superior in the Great Lakes (Grigorovich, Kelly, Darling, & West, 2008), yet appears to be widespread, since we found it in Lake Erie and throughout the Hudson River. Previous investigations described haplotypes QM–G as being widespread in the Dnieper River (where the quagga mussel natively occurs) and throughout its invasive range in Europe (Romania, Germany, France, and The Netherlands), as well as in Lake Huron and Lake Cayuga, NY (Marescaux et al., 2016). That haplotype was not previously known from Lake Erie, where Marescaux et al. (2016) surveyed just ten individuals. The veliger larvae collection and HTS analysis approach used here is much more likely to elucidate the presence of rare haplotypes, such as these, due to the tremendous number of larvae sampled and analyzed.

### 4.1 The present approach versus other eDNA techniques

Some previous studies found that qPCR is a useful tool for estimating the number of dreissenid mussel sequence copies in a plankton or environmental sample (Amberg et al., 2019; De Ventura et al., 2017; Ram et al., 2011). However, those each required two separate markers (one for zebra mussels and another for quagga mussels) and neither discerned population genetic variability. Ram et al. (2011) used mt16S RNA for zebra mussel and mtDNA COI for quagga mussel, with the two targeted regions differing by 181 nt in length. Amberg et al. (2019) designed COI primers for the zebra mussel, with their future aspirations stated to develop a probe to differentiate quagga mussel eDNA. De Ventura et al. (2017) employed two overlapping regions of mtDNA COI, which had different primers and differed in length by 100 nt. Thus, their respective targeted regions would have differential amplification, and could degrade at different rates in the environment, rendering the two products of the two species not directly comparable. This likely would lead to inaccurate estimates of their relative abundances (see Dejean et al., 2011) in both methods. Two other qPCR assays for dreissenids (Gingera et al., 2017; Peñarrubia et al., 2016) lacked the ability to distinguish between the species. Egan et al. (2015) used light transmission spectroscopy to test water from the hulls of ships from Great Lakes harbors, and were able to detect eDNA from the quagga mussel, but not the zebra mussel, from localities where both were known to be present. Our approach overcomes such issues by using a higher‐resolution HTS metabarcoding approach, amplifying the same gene region for both species.

The present targeted metabarcoding HTS assay yields important intraspecific population‐level information for dreissenid mussels, beyond the scope of the earlier published methods. For example, the Klymus et al. (2017) 16S assay accurately differentiates between the two species, but that gene region evolves too slowly to provide population genetic resolution. Our study results here discerned haplotypic diversity of zebra and quagga mussels that was undetected by conventional sequencing of adults (Marescaux et al., 2016; Stepien et al., 2013). Similar intraspecific approaches using the mtDNA control region have been developed for the whale shark *Rhincodon typus* (Sigsgaard et al., 2016) and harbor porpoise *Phocoena phocoena* (Parsons et al., 2018), and with the cytochrome *b* region for the invasive silver carp *Hypophthalmichthys molitrix* (Stepien et al., 2019). Our assay is designed to accurately estimate haplotypic frequencies, as well as relative species proportions, which will be useful for elucidating invasion pathways, along with documenting population relationships across temporal and spatial scales.

In summary, this diagnostic approach identifies and determines the relative representation of closely related species and their populations from plankton and eDNA, facilitating community‐ and population‐level detection and ecological comparisons. The new targeted HTS metabarcoding dreissenid assay yields key information about the relative proportions of inter‐ and intra‐specific variation. Tremendous numbers of individuals can be rapidly and simultaneously assessed, discerning and quantifying the relative abundances of species and their population‐level haplotypic diversity among locations. Present‐day morphological techniques for early detection of dreissenid invasions from plankton have been deemed insufficient for western U.S. reservoirs (Counihan & Bollens, 2017), with eDNA suggested as a useful alternative (Hosler, 2017). In comparison, our method, which jointly evaluates inter‐ and intra‐specific diversity of plankton and eDNA water samples, allows for analysis of the temporal and spatial trajectories of dreissenid invasions, including potential displacements of zebra mussel populations by quagga mussels. Our assay and approach thus show broad and widespread application as an effective monitoring and research tool for understanding biological invasions and resultant ecological changes.

## CONFLICT OF INTEREST

None Declared.

## AUTHOR CONTRIBUTIONS

C.A.S. conceived the study, wrote the grant, and obtained the funding. N.T.M. conducted sample collection, laboratory analyses, and developed the figures and tables. Data analysis, manuscript drafting, and manuscript revising and editing were done together by N.T.M. and C.A.S.

## Data Availability

New haplotypes discerned in the present study are deposited in NCBI GenBank (accession numbers MK358469–70). Raw sequence data are provided in the NCBI GenBank repository under BioProject PRJNA513276 with accession numbers SAMN10699433–SAMN10699476.

## APPENDIX A

**Table A1:** Single nucleotide polymorphisms (SNPs) that differentiate zebra mussel (ZM; *Dreissena polymorpha*) and quagga mussel (QM; *D*. *rostriformis*) haplotypes (lettered) for the COIA region (from NIH NCBI GenBank and our records). Haplotypes used for our mock communities are in ***bold italics***. Primer regions are shaded grey

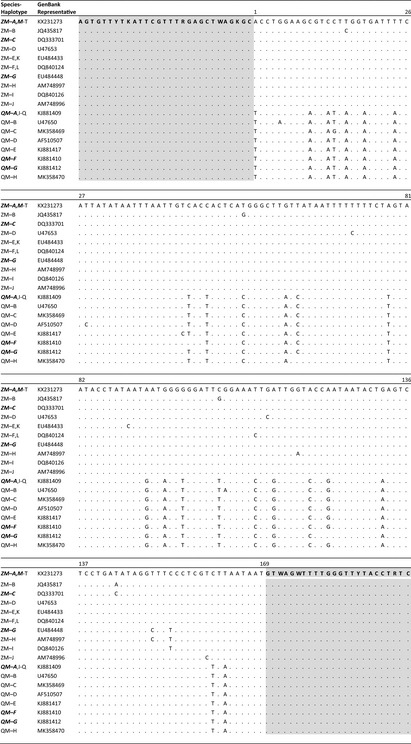

**Table A2:** Consensus sequences for six species of *Dreissena *and two closely related invasive *Mytilopsis *species for the COIA region from NIH NCBI GenBank and our records. Primer regions are shaded grey

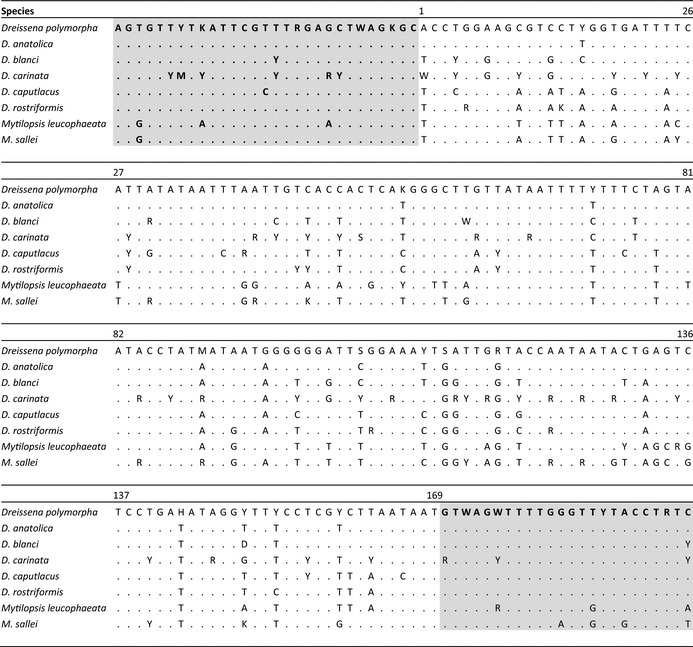

**Table A3:** Single nucleotide polymorphisms (SNPs) that differentiate zebra mussel (ZM; *Dreissena polymorpha*) and quagga mussel (QM; *D. rostriformis*) haplotypes (lettered) for the COIB region from NIH NCBI GenBank and our records. Haplotypes used for mock communities are in *bold italics*. Primer regions are shaded grey

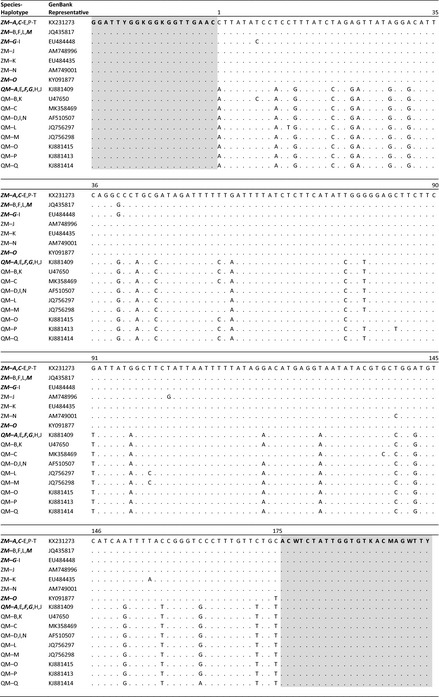

**Table A4:** Consensus sequences for six species of *Dreissena *and two closely related invasive *Mytilopsis *species for the COIB region from NIH NCBI GenBank and our records. Primer regions are shaded grey

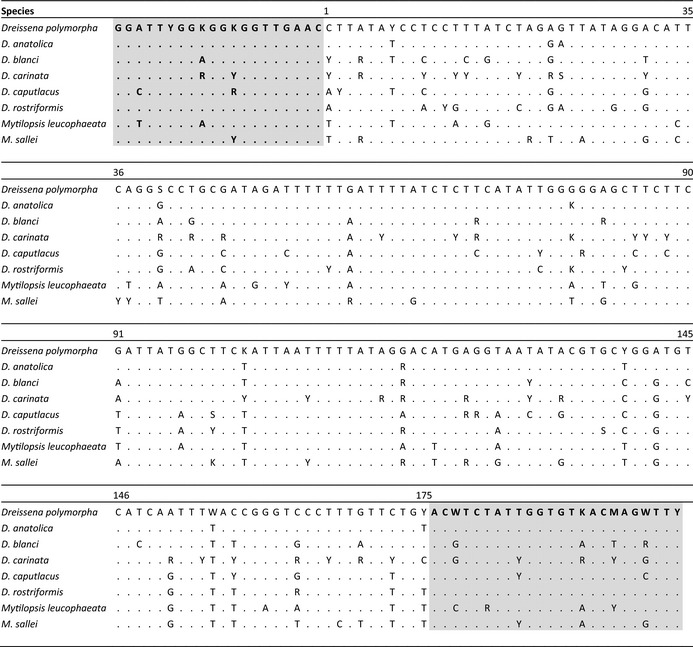

**Table A5:** Number of reads, merged reads, and exact match trimmed reads (called) for mock community, plankton, and water samples. *MC5A, MC2B, and MC7B were analyzed with the OBItools Python package, instead of the DADA2 pipeline, due to less sequencing depth*

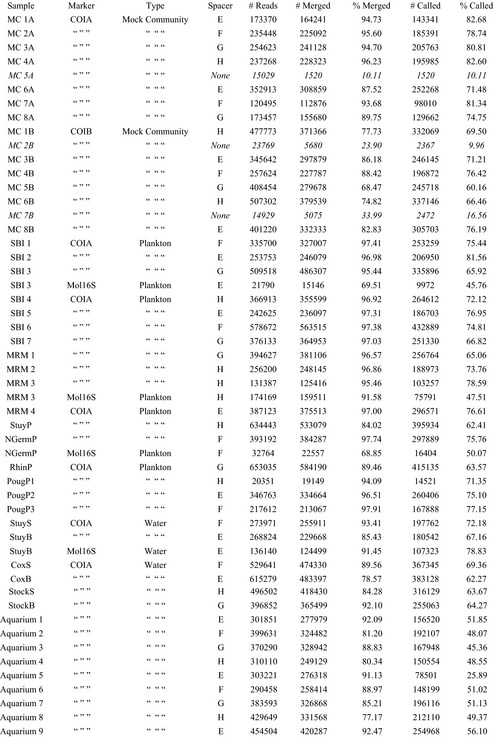

**Table A6:** Pairwise comparisons between environmental samples based on mtDNA COIA HTS results. (a) Pairwise Kulczynski Dissimilarity Index (KDI) between all samples and (b) Pairwise KDI value for the average proportions of each sampling group
